# Protein C in adult patients with sepsis: from pathophysiology to monitoring and supplementation

**DOI:** 10.1186/s44158-025-00243-0

**Published:** 2025-04-14

**Authors:** Irene Coloretti, Antonio Corcione, Gennaro De Pascale, Abele Donati, Francesco Forfori, Marco Marietta, Mauro Panigada, Paolo Simioni, Carlo Tascini, Pierluigi Viale, Massimo Girardis

**Affiliations:** 1 Anaesthesiology and Intensive Care Department, University Hospital of Modena, University of Modena, Reggio Emilia, Modena, Italy; 2 Department of Critical Care, AORN Ospedali Dei Colli, Naples, Italy; 3 Dipartimento Di Scienze Biotecnologiche Di Base, Cliniche Intensivologiche E Perioperatorie, Università Cattolica del Sacro Cuore, Rome, Italy; 4 Dipartimento Di Scienze Dell’Emergenza, Fondazione Policlinico Universitario A. Gemelli IRCCS, Anestesiologiche E Della Rianimazione, Rome, Italy; 5 Department of Biomedical Sciences and Public Health, Università Politecnica Delle Marche, Ancona, Italy; 6 Anesthesia and Intensive Care, Azienda Ospedaliero Universitaria Delle Marche, Ancona, Italy; 7 Dipartimento Di Patologia Chirurgica, Medica, Molecolare Ed Area Critica, Università Di Pisa. AOUP, Pisa, Italy; 8 Department of Hematology-Azienda Ospedaliero, Universitaria Di Modena, Modena, Italy; 9 Department of Anesthesia, Intensive Care and Emergency, Fondazione IRCCS Ca’Granda Ospedale Maggiore Policlinico, Milan, Italy; 10 Clinica Medica 1, Azienda Ospedale Università Di Padova, Padua, Italy; 11 Department of Medicine (DMED), University of Udine, Udine, Italy; 12 Infectious Diseases Clinic, ASUFC “Santa Maria Della Misericordia” University Hospital of Udine, Udine, Italy; 13 Department of Medical and Surgical Sciences, Alma Mater Studiorum, University of Bologna, Via Massarenti 9, 40138 Bologna, Italy; 14 Infectious Diseases Unit, Department for Integrated Infectious Risk Management, IRCCS Azienda Ospedaliero-Universitaria Di Bologna, Bologna, Italy

**Keywords:** Sepsis, Septic shock, Thrombosis, Disseminated intravascular coagulation, Activated protein C

## Abstract

Protein C (PC) plays a crucial role in modulating inflammation and coagulation in sepsis. Its anticoagulant and cytoprotective properties are critical in mitigating sepsis-induced coagulopathy, which is associated with high mortality rates. In sepsis, low levels of PC are associated with an elevated risk of multiple organ dysfunction and increased mortality. Routine monitoring of PC levels is not widely implemented but appears relevant in selected populations, such as patients with purpura fulminans, sepsis-induced coagulopathy (SIC), disseminated intravascular coagulopathy (DIC) or hyperinflammatory septic shock phenotypes. Treatment with PC has been limited to PC concentrate approved for paediatric use in congenital PC deficiencies and purpura fulminans, while the efficacy of PC supplementation in sepsis remains a subject of debate. Considering the physiological significance of PC and its role in sepsis pathophysiology, additional studies are necessary to fully elucidate its therapeutic efficacy in specific clinical settings.

## Background

The interplay between the immune response, coagulation, and endothelial function regulates the host response to infection, and the dysfunction in their interaction results in a dysregulated response to infection [1]. The inflammatory immune response in sepsis induces prominent activation of leukocytes, platelets, and endothelium, precipitating excessive activation of the coagulative system, frequently accompanied by impaired anticoagulant and fibrinolytic systems, culminating in sepsis-induced coagulopathy in at least a quarter of septic patients [2–5]. Sepsis-induced coagulopathy (SIC) is a clinically relevant complication of sepsis-induced organ dysfunction, and the progression to disseminated intravascular coagulation is associated with a high risk of mortality [6, 7].

Endogenous protein C (PC), a vitamin K–dependent serine protease synthesized predominantly in the liver, exhibits significant anticoagulant and cytoprotective activities, and thus plays a crucial role in the interplay between inflammation and coagulation. Numerous studies have demonstrated that in patients with sepsis, low levels of PC are associated with an elevated risk of multiple organ dysfunction and increased mortality rates. Consequently, PC has been considered a potential therapeutic target in critically ill patients for several decades, albeit with inconclusive results and lack of definitive evidence.

In this narrative review, we aimed to elucidate the basic mechanisms underlying coagulation disturbances in sepsis, examining the role of the PC pathway and its alterations in patients with sepsis, and evaluating the rationale for PC assessment and eventual supplementation in these individuals.

### Sepsis-induced coagulopathy

Proteinase-activated receptors (PARs) act as a link between immune response and coagulation, interacting with Toll-like receptors (TLRs) to enhance the release of pro-inflammatory mediators and induce the production of tissue factor (TF), an initiator of the coagulation cascade [8–10]. Interleukin- 1 (IL- 1) can upregulate the expression of TF in endothelial cells, monocytes, and other cell types, leading to an increased procoagulant state [11]. Immune-thrombosis (or thromboinflammation) refers to thrombin generation and microthrombus formation, contributing to pathogen recognition and containment activated primarily by the innate immune response [12].

Alongside this, it has been demonstrated that thrombin can directly activate surface pro-IL- 1α on macrophages and activated platelets, while tissue factor, a potent thrombin activator, colocalizes with pro-IL- 1α in the epidermis. Thrombin-cleaved IL- 1α was detected in humans during sepsis, highlighting the relevance of this pathway for both normal physiology and the pathogenesis of inflammatory and thrombotic diseases [13].

Coagulation factors such as thrombin can activate immune cells and increase the production of pro-inflammatory cytokines. Fibrin, the main component of blood clots, acts as a scaffold for immune cells and promotes their recruitment and activation at site of infection [14].

In clinical practice, SIC is often considered synonymous with Disseminated Intravascular Coagulation; however, these two terms refer to different stages of coagulopathy in patients with sepsis. SIC is a non-overt disseminated intravascular coagulation (DIC), also described as systemic intravascular coagulation, but without gross consumption of coagulation components, and is characterized by a procoagulant state in the early stages of sepsis [15]. Recently, a definition of sepsis-induced coagulopathy was introduced to identify patients at an earlier stage when changes in coagulation status are still reversible [16] by targeting excessive immune activation, thrombin generation, and endothelial dysfunction [17]. Furthermore, the ISTH released a set of simplified diagnostic criteria designed to detect SIC in the early phase of sepsis before progression to overt DIC [18]. SIC includes a Sequential Organ Failure Assessment (SOFA) Score of > 2, platelet count, and prothrombin time, making this assessment easily performed at the bedside, as the SOFA score is traditionally used to evaluate the severity and extent of organ failure in septic patients. The SOFA is composed of scores from six organ systems, graded from 0 to 4 according to the degree of dysfunction/failure. In the Sepsis- 3 definition, organ dysfunction was identified and quantified using the SOFA score as it is well established, widely used, easily implemented and performs reasonably well in the early prediction of outcome in ICU patients with infection [19].

The prevalence and mortality of SIC in sepsis (using the Sepsis- 3 definition) were evaluated in a secondary analysis of two RCTs [6], in which the prevalence of SIC was 22.1% and 24.2%, respectively. The 90-day mortality rate of patients with SIC in the HYPRESS study (sepsis without shock) was twofold higher than that of patients without SIC. Although a small population (15 patients in the SIC group and 28 patients in the no-SIC group died), the study concluded that SIC is associated with higher morbidity and mortality and should be diagnosed and managed to reduce sepsis-associated organ injury.

In the last edition of Surviving Sepsis Campaign guidelines, the role of altered coagulation and SIC recognition is poorly emphasized, and the chapter “anticoagulation’’, referring to possible therapeutic interventions, was omitted from the latest edition of the guidelines [20]. In contrast, the latest Japanese Sepsis guidelines of 2020, encourage early detection of DIC and weakly recommend the use of anticoagulants for sepsis-associated DIC [21].

### Protein C synthesis and mechanisms of action

PC is synthesized predominantly in the liver, but also in the epididymis, kidney, lung, brain, and male reproductive tissue [22] as a single polypeptide chain of 461 amino acids [23]. PC is multimodular and contains structural elements that are similar to those of other vitamin K-dependent coagulation proteins. Following transcription, γ-carboxylation is essential for the efficient secretion of PC and for imparting its pleiotropic properties, which include anticoagulant and cytoprotective effects. However, to fully realize these pleiotropic effects, PC must be activated to form activated protein C (aPC). The conversion of PC to aPC results from the cleavage of an Arg^169^-Leu^170^ peptide bond, which releases a dodecapeptide from the heavy chain. This reaction is enhanced by binding of thrombin to thrombomodulin [24]. After its production, the half-life of PC in the bloodstream is 6–10 h [25].

PC exerts its anticoagulant properties by inactivating the factors Va and VIIIa, which are important cofactors of the coagulation cascade. These events are enhanced by the presence of Ca^2+^, phospholipids, and protein S as cofactor [26]. Other functions of aPC in haemostasis rely on its ability to downregulate thrombin and suppress the activation of thrombin activatable fibrinolytic inhibitor (TAFI), thus indirectly promoting fibrinolysis [27]. Fibrinolysis is also enhanced by aPC through inhibition of plasminogen activator inhibitor- 1 (PAI- 1) [28].

Protein C also exhibits cytoprotective properties, including anti-inflammatory and anti-apoptotic activities, and preserves endothelial barrier function. Its anti-inflammatory properties are mediated through the suppression of transcription of nuclear factor-κB (NF-κB) subunits, thereby inhibiting the expression of downstream NF-κB target genes. This process consequently impedes the tumour necrosis factor (TNF)-dependent induction of adhesion molecules, such as E-selectin, ICAM- 1, and VCAM- 1. Concomitantly, aPC downregulates TNF-α–induced binding of monocytes [29]. In addition, aPC was found to inhibit lipopolysaccharide (LPS)- and interferon-γ–related production of several proinflammatory cytokines in monocytes [30], and to increase the production of anti-inflammatory cytokines such as interleukin- 10 (IL- 10) and transforming growth factor-β [31]. In vitro studies have demonstrated that aPC can block neutrophil and eosinophil migration when stimulated by chemoattractants [32, 33].

Antiapoptotic activity relies on the ability of aPC to modulate antiapoptotic transcription profiling, upregulating the antiapoptotic protein, B-cell lymphoma- 2, the endothelial survival factor eNOS, and the inhibitor of apoptosis genes, and the suppression of genes promoting apoptosis, such as calreticulin and TRMP- 2 [29]. Moreover, aPC treatment blocks the induction of apoptosis in several cell lines and inhibits TNF-α-stimulated apoptosis [34–36].

### Protein C in sepsis

The role of the PC pathway in sepsis has been widely investigated and demonstrated to be crucial in the response to invading organisms [14, 37] (Table 1). The activation of Toll-like receptor 4 (TLR4) on monocytes mediated by pathogens or immunostimulatory pathogenic agents, such as endotoxin [38], leads to the activation of coagulation through the upregulation of tissue factor (TF) [39] and enhances cytokine production, especially TNF-α, IL- 1, IL- 6, and IL- 8 [40]. These cytokines inhibit the nuclear transcription of thrombomodulin (TM) and EPCR, and promote receptor shedding and cleavage of TM from the endothelial surface mediated by neutrophil elastase, with the overall effect of diminishing the capacity of the endothelium to activate PC [41]. In addition, TNF-α and IL- 1 primarily inhibit PC production in the liver and other organs by blocking mRNA transcription [42]. Additionally, low levels of protein C induced by inflammation lead to higher levels of available PAI- 1 and higher levels of thrombin, thus enhancing the activation of TAFI and inhibiting fibrinolysis [43]. Moreover, higher levels of thrombin and other coagulative proteases influence proinflammatory signalling through protease-activated receptors, thus making this process self-sustaining and triggering a vicious cycle [44]. As a result, the overexpression of cytokines in sepsis and septic shock promotes the coagulation pathway by inhibiting anticoagulant components such as protein C. This process is further amplified by reduced fibrinolysis, associated with low levels of aPC.

**Table 1:** Effects of endogenous protein C, experimental models, and possible clinical relevance in patients with infection

Tissue	Effects	Experimental models	Clinical relevance
Cellular	a) Cytoprotective activity through PAR- 1 b) Anti-apoptotic activity downregulating p53 and Bax	In vitro	**
Vascular	a) Endothelial barrier stabilization through up-regulation of sphingosine kinase- 1	In vitro	**
Coagulation	a) Anticoagulant activity through inactivation of FVa and FVIIIa on negatively charged phospholipid membranes	Animal Human	****
Immune	a) Reduced production of inflammatory cytokines through NF-kB inhibition b) Reduced expression of cell adhesion molecules such as ICAM- 1 and VCAM- 1 → Inhibition of leukocyte recruitment	Animal	***

^**^Valuable

^***^Important

^****^Relevant

The endothelium plays a crucial role in the septic response [45] and PC system regulation, as the production of inflammatory mediators such as TNF-α and thrombin in the bloodstream has significant downstream effects on endothelial cells. Primarily, thrombin alters the integrity of the endothelial barrier [46], likely through the downregulation of tight junction proteins and the subsequent rearrangement of the cytoskeleton [47]. This endothelial activation triggers various effects that regulate the septic response, including extravasation of inflammatory leukocytes in tissues. The production and secretion of PAI- 1 by the activated endothelium results in the inhibition of fibrinolysis and upregulation of coagulation. Some studies have suggested that leukocytes may be the major targets for the protective effect of aPC on the endothelium in severe sepsis. In fact, it has been demonstrated that aPC binds to leukocyte integrins, thereby inhibiting the migration of neutrophils into tissues [48]. Moreover, results of several studies have indicated that the EPCR-dependent endothelial protective activity of APC is mediated through crosstalk with other G- and non-G-protein coupled receptors [33, 49, 50]. Finally, some studies have revealed that when EPCR is occupied by protein C, the cleavage of PAR- 1 by thrombin elicits only protective signalling responses in endothelial cells [51].

Many of these effects have been observed in subjects with genetic deficiencies in PC, resulting in very low endogenous protein levels. In studies conducted in mice, were demonstrated hypercoagulable and hyperinflammatory patterns [52, 53]: following an LPS challenge, low endogenous levels of PC make mice susceptible to early-onset DIC, thrombocytopenia, hypotension, organ damage, and decreased survival. Moreover, these low-PC mice exhibited a heightened inflammatory response, which was significantly less pronounced in wild-type cohorts. Symptomatic heterozygous deficiencies in humans can result in deep vein thrombosis and pulmonary embolism. Homozygous PC deficiencies are rare and associated with fatal systemic disseminated intravascular thrombosis, purpura fulminans, its cutaneous manifestation [54]. Evidence from these observations makes it clear that the PC pathway, thought to be merely a part of the haemostatic system, has emerged as a key mediator in inflammatory pathways. The PC pathway acts at the intersection of coagulation and inflammation and plays an important role in tissue injury and damage associated with acute and chronic inflammation.

### Protein C levels and monitoring

At birth, the natural anticoagulants are reduced with the notable exception of alpha- 2-macroglobulin, which is increased [55]. For this reason, in neonatal period PC levels are low, typically less than 50% of adult normal values [55–58], and gradually increase, reaching adult levels by 6–12 months of age [56, 59]. Additionally, PC is present in a'fetal'form at birth [59, 60], though its physiological differences remain unclear. Preterm infants exhibit even lower levels at birth [61], while small-for-gestational-age (SGA) neonates are reported to have a certain degree of resistance to activated PC [62]. In adult patients normal values of PC plasma activity have been assessed from 70 to 150% [63].

PC levels can be measured in plasma by activity and antigen assays. Functional tests for PC activity are mainly based on two methods: (1) the aPTT derived method (coagulometric or anticoagulant) in which the prolongation of the clotting time after activation of PC by Protac™ (Pentapharm, DSM biomedical) is proportional to the amount of PC present in patient’s plasma; (2) in the chromogenic (amidolytic) method protein C activity is detected by means of a specific chromogenic substrate which is cleaved by aPC after activation of PC by Protac™ [64]. The former is also more sensitive to the presence of gamma-carboxylation forms of PC, the latter better explores the catalytic activity of PC, once activated, towards its specific chromogenic substrate. The concentration of PC present in patient’s plasma can be detected by an ELISA antigen assay using catching monoclonal antibodies against PC and detecting anti-PC antibodies bound to horseradish peroxidase. In general, PC functional activity mirrors PC antigen levels. However, in case of liver dysfunction there can be hypocarboxylated forms of vitamin K-dependent factors including PC and lower functional levels can be detected by coagulometric as compared to amidolytic activity method, as in cases of congenital type II PC deficiencies (Table 2). Thus, in such a situation the correct selection of the method used for the evaluation of PC levels in plasma is important. These features are well known in patients with inherited PC defects whose PC activity levels may vary depending on the type of functional method used [65] (Table 2).

**Table 2:** Characteristics of congenital and acquired Protein C deficits

Type of deficiency	Mechanisms of deficiency	Antigen	Chromogenic activity	Coagulometric activity
Congenital deficiencies				
I	Quantitative or true deficiency (75% of patients)	↓	↓	↓
II	Qualitative or dysfunctional (IIa 23,75% and IIb 1.25% of patients)	N	↓ (IIa) N (IIb)	↓
II	Quantitative and qualitative	↓	↓↓	↓↓
Acquired deficiencies				
Liver diseases Vitamin K deficiency AVK treatment DIC Sepsis Chemotherapy with L-asparaginase Nephrotic syndrome Solid malignancies	Reduced synthesis/increased clearance	↓	↓	↓
Antibodies against PC	Mainly inhibitors	N	↓	↓
Factors potentially interfering with results				
Pregnancy		–	↑ (early)	–
DOAC		–	-	False ↑
Clotted/activated samples		–	False ↑	False ↓
Lupus anticoagulant (aPTT-based, not dRVVt)		–	–	False ↑
Factor V Leiden/Factor VIII > 200%		–	–	False ↓ (with aPTT-based assays)
Increased FVIII activity > 200%			–	False ↓ (with aPTT-based assays)

Modified from: Dinarvand P, Moser KA. Protein C deficiency. Arch Pathol Lab Med 2019; 143:1281- 1825; Cooper PC, Pavlova A, Moore GW, Hickey KP, Marlar RA. Recommendations for clinical laboratory testing for protein C deficiency, for the subcommittee on plasma coagulation inhibitors of the ISTH. J Thromb Haemost. 2020;18(2):271–277

*AVK* anti-vitamin K, *DIC* disseminated intravascular coagulation, *PC* protein C, *aPTT* activated partial thromboplastin time

Previous studies have demonstrated that more than 80% of patients with severe sepsis have a baseline PC level below the lower normal cut-off, a reduction in endogenous PC levels [66] due to increased consumption, decreased protein synthesis in the liver, and proteolytic degradation by neutrophil elastase [67–70]. Given the aforementioned pleiotropic effects of PC, it seems reasonable to hypothesize that low levels may affect the clinical course of sepsis and septic shock [71–76] (Table 2). To date, routine measurement of PC is not commonly used in sepsis [20] and has only been reported in a few clinical studies. Low protein C activity has been demonstrated to be a good predictor of the degree of organ dysfunction in an observational study conducted on 743 septic patients in intensive care unit (ICU) [74]. The authors demonstrated that reduced PC activity correlated with specific components of the SOFA score, including platelet count, liver function, and circulation. Other studies have focused on the relationship between PC activity and acute kidney injury (AKI), revealing that PC activity decreases significantly according to AKI severity [71–73]. A recent meta-analysis, including 12 studies, demonstrated that PC levels were significantly higher in sepsis survivors than in non-survivors and in patients with sepsis without disseminated intravascular coagulation [75]. Considering the physiological role of PC, the pathophysiology of sepsis, and the observed data, it seems reasonable to consider the measurement of PC levels useful to better define the coagulative and inflammatory status of patients with sepsis and septic shock.

There are assays that allow to quantify circulating aPC levels with high sensitivity and accuracy and can be performed in specialized laboratories. These assays can be classified into two groups: those that capture aPC and directly measure its enzymatic activity [77–80], and those that use heparin during blood sampling and quantify the aPC-Protein C inhibitor (PCI) complex [81, 82]. The main advantage of the first group of assays is that they measure free circulating activated PC levels, while those assays in the second group measure free aPC bound to its inhibitor, PCI, and aPC. However, these assays also reflect the concentration of circulating aPC quite accurately, since the levels of aPC:PCI complexes circulating at the time of sampling were much lower than the aPC:PCI levels formed from the free aPC present at sampling [82, 83]. They also measured only the circulating aPC that is active, since its activity is necessary to inactivate and complex with its inhibitor, PCI.

### Monitoring of protein C: which patient?

Although PC is not routinely measured in clinical practice, it appears to be particularly relevant in three septic populations with potential coagulation disorders: those with purpura fulminans, those with septic shock and positive SIC/DIC, and those with septic shock exhibiting a hyperinflammatory phenotype.

Purpura fulminans is most frequently seen in children presenting with severe septic shock due to meningococcemia [84]; otherwise, purpura fulminans also occurs in the scenario of homozygous or double heterozygous PC deficiency [85]. Indeed, purpura fulminans may be described as the clinical manifestation of a severe PC deficiency and acute PC pathway failure [86]. Consequently, PC substitution in patients with purpura fulminans has been adopted with promising results [87–90]. Measurement of PC levels to assess diagnosis and severity of its deficiency and eventually to initiate the treatment and tailor the dosage should be considered in patients with purpura fulminans.

Similarly, it seems useful to measure PC levels in patients with septic shock and signs of SIC/DIC as PC plays a role in the interaction between immune system and coagulation pathways. Animal studies demonstrated that low endogenous levels of PC were associated to early-onset DIC in sepsis models and, in humans, symptomatic heterozygous deficiencies can result in fatal systemic disseminated intravascular coagulation [54].

**Fig. 1:**
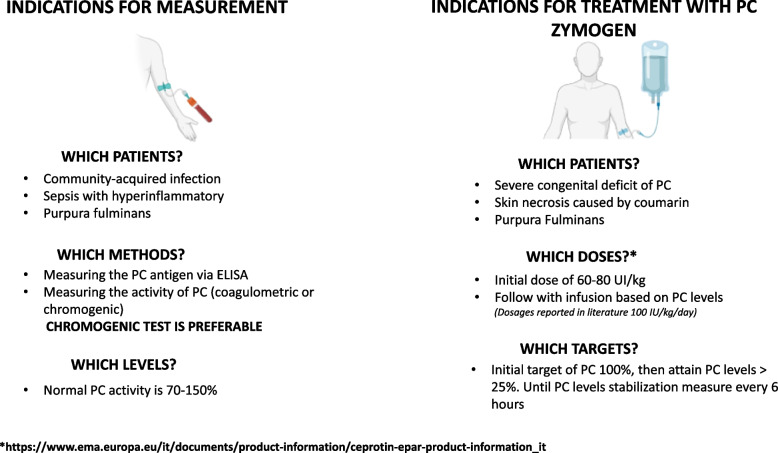
Proposed clinical scenarios for measuring protein C in critically ill patients and proposed indications for PC concentrate treatment in specific subpopulations

The hyperinflammatory phenotype of septic shock is referred to the early phases of sepsis, in which the pro-inflammatory response predominates. This phase is characterized by the massive production of proinflammatory cytokines associated with inappropriate anti-inflammatory response [91]. This may quickly result in multiple organ dysfunction, overwhelming shock, and death [92]. In this phase, functional impairment of the endothelium plays a key role, and as this mechanism proceeds, may lead to coagulopathy (SIC/DIC). In these patients, early measurement of PC at the onset of shock may help predict the development of SIC/DIC and potentially guide the use of preventive strategies for these complications, which are associated with an increased risk of mortality (Fig. 1).

### Treatment with protein C

When considering treatment with protein C, two types of preparations have been proposed: recombinant activated protein C and the inactive zymogen form, which requires activation within the patient’s system. However, following the market withdrawal of the recombinant activated protein C formulation (drotrecogin alfa activated, DAA) in 2011—based on preliminary results from the PROWESS-SHOCK follow-up trial [93]—its use was discontinued, shifting the focus of clinical trials toward the inactive zymogen formulation. Otherwise, the effectiveness of infused inactivated PC in mitigating the effects of septic shock on coagulation relies on its successful conversion to aPC in vivo. While a previous study demonstrated that PC concentrate is converted to aPC in asymptomatic individuals, its activation in patients with PF and septic shock, who exhibit a severe coagulation pathway derangement, had yet to be established. Recently, a study suggested that this activation process may be impaired in such patients [94]. This is attributed to the thrombomodulin-protein C (TM-PC) system, which is believed to play a pivotal role in disease pathogenesis [95, 96]. According to this model, the early loss of TM impairs the activation of both endogenous and therapeutically administered protein C. However, some studies have indicated that infused PC can be effectively converted to aPC in patients with PF, suggesting that supplemental protein C may be beneficial even in cases of TM-PC pathway failure [87, 96].

### Purpura fulminans

Purpura fulminans is a life-threatening condition associated with infection characterized by the association of a sudden and extensive purpuric rash together with an acute circulatory failure [97]. Purpura fulminans accounts for less than 0.5% of the cases of septic shock. *Neisseria meningitidis* and *Streptococcus pneumoniae* are the leading causative bacteria [97] and, although these pathogens are highly susceptible to available antibiotics, mortality and morbidity of purpura fulminans remain very high [98]. This occurs because the invading pathogen triggers a rapid activation of the immune and coagulation systems. Once initiated, this process becomes self-sustaining, causing damage even after the pathogen has been eradicated. Experimental models show that the specificity of meningococcal purpura fulminans compared to other diseases causing disseminated intravascular coagulopathy is mainly caused by the interaction between Neisseria Meningitidis and endothelial cells, with subsequent vascular damages [99, 100]. The alterations of the endothelium induce the development of an acquired PC deficiency with possible implications on both local coagulation and inflammation [95]. Interestingly, purpura fulminans also occurs in the rare scenario of homozygous or double heterozygous protein C deficiency [85]. Indeed, the clinical manifestation of a severe PC deficiency in the form of purpura fulminans has resulted in the description of purpura fulminans closely related to acute PC pathway failure [86]. Consequently, many intensivists have used PC substitution in patients with purpura fulminans, with promising results both in children and adults [87–90, 101, 102] (Table 3). First small observational studies were conducted on pediatric populations [87, 88, 103] with PF caused by infectious source, demonstrating positive effects on predicted morbidity and mortality reduction, and in restoring haemostatic balance and microcirculation integrity. In 2003, a double-blind RCT randomized 40 children with PF and septic shock to receive intravenous placebo (albumin human 1%) or 50 IU/kg, 100 IU/kg, or 150 IU/kg PC concentrate given as an intravenous bolus every 6 h during the first 72 h after study entry and every 12 h thereafter, up to a maximum total treatment period of 7 days [90]. Authors concluded that PC concentrate is a safe and effective treatment for children with purpura fulminans and meningococcal septic shock, resulting in dose-dependent increases in aPC plasma levels and the correction of coagulation disturbances. A subsequent retrospective multicenter study included 94 children with PF and septic shock, primarily caused by *Neisseria meningitidis* (79.8%), who received treatment with protein C concentrate. The study demonstrated the absence of major bleeding events, with only cases of epistaxis reported. Additionally, a lower incidence of dermatoplasty and limb amputation was observed, potentially attributable to the beneficial effects of protein C on microcirculation. Considering adult patients, only small observational studies and case-series have been published [102, 104, 105], suggesting that PC concentrates in PF might be a useful treatment especially in limiting the extent of tissue necrosis. Interestingly, a case report published in 2018 [105] examined the kinetics of TM loss during PF, which is thought to impair the conversion of PC concentrate into activated PC. The authors found that TM loss was not an early event, occurring only after 72 h. This finding suggests that supplemental PC administration may remain effective even in patients who initially present with apparent failure of the TM-PC pathway.

**Table 3:** Summary of evidence on the use of PC concentrate in Purpura Fulminans

Reference	Design	Population	Dose	Main findings
Ettingshausen, 1999 [78]	Prospective observational	• 8 infants or adolescents (age 0.2 to 18.25 years) • meningococcal sepsis associated with PF • Overt DIC with low PC activity	Loading dose of 80 to 120 IU/kg followed by 50 IU/kg up to 6 times per day	• Correction of hemostasis (↑PC and ↓PAI- 1) and improvement of microcirculation in all patients • No adverse effect
White, 2000 [77]	Prospective observational	• 36 patients with mean age of 12 years (from 0.3 to 72) • Severe meningococcal septicemia, PF, and multiorgan failure	Loading dose of 100 IU/kg and a continuous infusion of 10 IU/kg/h. Then dose was adjusted to maintain a plasma PC level of 80–120 IU/mL	Reduction in predicted morbidity (Glasgow Meningococcal Septicemia Prognostic Score) and mortality
Rintala, 2000 [94]	Case series	• 12 adult patients • PF, overt DIC and sepsis	No loading dose, bolus of 100 IU/kg every 6 h	Amputations were necessary in two patients. The hospital mortality was 5 out of 12 (42%)
De Kleijn, 2003 [80]	Double-blind phase II RCT	• 40 children (age 1 month to 18 years) • Septic shock with petechial rash and/or PF	Intravenous placebo (albumin human 1%) or 50 IU/kg, 100 IU/kg, or 150 IU/kg Treatment was a bolus every 6 h for the first 72 h and every 12 h thereafter, up to a maximum treatment period of 7 days	• PC levels remained ↑ 6 h after the bolus in all treated patients vs no change in placebo • AUC for PC correlated with the dose of PC Concentrate • Cumulative dose of 600 IU/kg/day is necessary for sustained protein C activation • Activation of PC occurred in 27 of 28 treated patients
Schellongowski, 2006 [92]	Case series	• 8 adult patients (mean age 33.5) • Sepsis and PF, 5 had SS and in 50% caused by Neisseria Meningitidis	• 5 patients received loading bolus of 100 U/kg, then level-controlled continuous infusion starting at 10 U/kg/h • 3 patients received 100 U/kg bolus every 6 h	• Achievement of values of plasma aPC activity > 100% in all patients regardless of the infusion regimen • DIC could be controlled in all but one patient with first signs of improvement as an increase of fibrinogen and antithrombin levels, a decrease of D-dimer levels, • No major adverse bleeding events
Veldman, 2010 [91]	Retrospective multicentered observational	• 94 pediatric patients (mean age 2.46 years) • PF due to sepsis, Neisseria meningitidis 79.8% patients	• Treatment for median 33 h with median dose of 100 IU/kg/day • Bolus every 4 to 6 h in 78 patients, and as a bolus followed by continuous infusion in 16 patients	• ↑ time to treatment in non-survivors vs survivors (median 8.6 vs. 4 h, *p* = 0.03) and also ↑ in patients who had amputations • Plasma PC levels ↑ from 27 to 71% under treatment • Non-survivors had ↓ PC plasma levels (*p* < 0.05) • 4 adverse events in 3 patients, none severe
Piccin, 2014 [93]	Retrospective observational	• 30 children (median age 2 years) • Diagnosis of sepsis with 70% Neisseriae Meningitidis sepsis, 3% Streptococcus Pneumoniae sepsis, 27% haematologic/solid malignancies	Loading bolus of 100 IU/kg was followed by continuous infusion at 15 IU/kg/h Target PC activity of 100%, then maintenance phase to maintain a PC level of > 25%	• Overall survival was 75% at 6 months • Patients with Neisseria meningitides sepsis (*n* = 21) had overall survival of 90% vs 37% in those with chemotherapy-induced neutropenia (*p* = 0.011) • No hemorrhagic events occurred
Bendapudi, 2018 [95]	Case report	• Adult 39 years male • PF due to Capnocytophaga canimorsus bacteremia	Initial bolus of PC (100 IU/kg) and AT concentrate (3100 U), these agents were readministered every 12 to 24 h through day 10 to maintain values.70%	• Infused PC rapidly converted to aPC • TM protein persisted at the endothelial surface for up to 72 h

*PF* purpura fulminans, *DIC* disseminated intravascular coagulation, *PC* protein C, *PAI- 1*- plasminogen activator inhibitor- 1, *AUC* area under the curve, *AT* antithrombin

Overall, these studies suggest that treatment with PC can normalize plasma levels, lower markers of SIC/DIC and possibly reduce morbidity and mortality (Table 3). At present, PC concentrate has an off-label indication but is reimbursed by AIFA for children with meningococcal sepsis and/or purpura fulminans (Fig. 1). The dosage recommended by manufacturers is an initial bolus of 60–80 IU/kg, followed by dosage adjustments based on PC activity measurement via chromogenic assay to achieve a plasma PC activity of > 25% [106].

### Sepsis

Systemic inflammation characterizing host invasion by pathogens in sepsis is responsible for systemic coagulation activation with intravascular procoagulant phenotypic changes. As mentioned, in nearly 20% of patients [6], this inflammatory response disrupts the coagulation balance through many mechanisms, resulting in SIC, which may progress to overt DIC, leading to increased mortality risk [6]. These conditions are characterized by widespread activation of leukocytes, platelets, and endothelium, with excessive activation of the coagulative pathways, often combined with defective anticoagulant and fibrinolytic systems, resulting in widespread thrombosis and consumptive coagulopathy [2–4]. For these reasons, different anticoagulant treatments were proposed to manage SIC and DIC occurrence in sepsis [107], but the benefits and harm caused by these anticoagulants remain unclear [20, 108].

Based on the rationale of the documented deficiency of protein C in sepsis and its pleiotropic effects previously described, recombinant human-activated PC has been proposed for treatment of sepsis and septic shock with or without SIC or DIC. PC administration was demonstrated to inhibit thrombosis, promote fibrinolysis, and exert many anti-inflammatory properties and endothelial barrier protection functions [109, 110]. Drotrecogin alpha activated (DAA), a recombinant aPC, was approved in 2001 for the treatment of severe sepsis based on the results of the landmark PROWESS trial [69]. In this double-blind, multicenter RCT patients with severe sepsis were enrolled and assigned to receive an intravenous infusion of either placebo or drotrecogin alfa activated. Authors found that treatment significantly reduced mortality, D-dimer levels and inflammation markers levels (IL- 6). Otherwise, they state that treatment with aPC may be associated with an increased risk of bleeding. Further secondary analysis was conducted by dividing patients enrolled in the PROWESS study in subpopulations based on mortality risk at baseline [111]. The authors found that the administration of drotrecogin alfa (activated) to patients with severe sepsis was associated with a significant survival benefit that tended to increase with higher baseline severity. Otherwise, the initial success of this treatment could not be replicated in patients with a low risk of death or in children with severe sepsis [112, 113]. In October 2011, Eli Lilly withdrew DAA from the market based on the preliminary results from the follow-up PROWESS-SHOCK trial that showed no mortality benefit at 28 days [93]. This double-blind, placebo-controlled, multicenter RCT randomized 1697 patients with septic shock to receive aPC or placebo for 96 h. The authors failed to demonstrate any benefit in death from any cause at 28 days. Of note, results from this trial have been largely debated. First of all, statistical power for mortality detection was < 80%, secondarily, mortality was lower than expected and then that in PROWESS. Few studies were published on the use of PC zymogen in patients with septic shock, reporting in some observational studies the safety and potential efficacy of this treatment [114, 115]. In 2016 Zangrillo et al. performed a double blind RCT including adult patients with severe sepsis or septic shock and high risk of death and of bleeding (APACHE II greater than 25, extracorporeal membrane oxygenation or DIC) [116]. Patients were randomized to receive PC zymogen (50 IU/kg in 20 min followed by continuous infusion at 3 IU/kg/h) or equivalent volume normal saline as placebo for 72 h. The study was stopped early for concomitant safety issue: ICU mortality was 79% (15 patients) in the PC zymogen group vs 39% (7 patients) in the placebo group (*p* = 0.020), and 30-day mortality was 68 vs 39% (*p* = 0.072). Otherwise, some limitations have to be issued: though patients with DIC were included, the proportion was only 21.6% of the population; patients included had extremely high baseline mortality risk (65%); it might had been more useful to show changes in activated PC activity rather than activated partial thromboplastin time and prothrombin time, as the main effect of PC is exerted on thrombomodulin. Some authors addressed that it may be too early to conclude that PC zymogen is not effective in all cases of sepsis, especially when SIC or DIC are present [117].

After these trials, there remains significant debate regarding these conflicting findings. Since the withdrawal of DAA, treatment with PC zymogen has been limited to PC concentrate only approved for pediatric use in congenital PC deficiencies and in purpura fulminans (Fig. 1).

## Conclusions

PC plays a key role in modulating inflammation and coagulation in sepsis. Routine monitoring of PC levels, although not routinely implemented, can be useful in specific populations, such as patients with purpura fulminans, SIC/DIC, or hyperinflammatory septic shock phenotypes. Because of the scarce available evidence, a large debate persists regarding the efficacy of PC supplementation in sepsis, though PC treatment is currently approved, in USA and EU, only for congenital deficiencies and purpura fulminans.

Nevertheless, given the physiological role of PC and its involvement in sepsis pathophysiology, further research is warranted to better define its therapeutic potential, in well-selected patient groups, with the aim at enhancing major clinical outcomes.

## Data Availability

No datasets were generated or analysed during the current study.

## Abbreviations

PC: Protein C
SIC: Sepsis-induced coagulopathy
DIC: Disseminated intravascular coagulopathy
PARs: Proteinase-activated receptors 1
TLRs: Toll-like receptors
TF: Tissue factor
IL: Interleukin
SOFA: Sequential organ failure assessment
SSC: Surviving sepsis campaign
TAFI: Thrombin activatable fibrinolytic inhibitor
PAI- 1: Plasminogen activator inhibitor-1
NF-kB: Nuclear factor-κB
TNF: Tumor necrosis factor
LPS: Lipopolysaccharide
TM: Thrombomodulin
EPCR: Endothelial protein C receptor
TAFI: Thrombin-activatable fibrinolysis inhibitor
AKI: Acute kidney injury
aPTT: Activated partial thromboplastin time
PF: Purpura fulminans
DAA: Drotrecogin alpha activated
